# Higher prevalence of short stature and excess weight in children with sleep disorders

**DOI:** 10.3389/fendo.2025.1680187

**Published:** 2025-11-19

**Authors:** Inbal Halabi, Dana Hadar, Hilla Cohen, Yardena Tenenbaum Rakover, Giora Pillar

**Affiliations:** 1 Pediatric Endocrine Unit, Carmel Medical Center, Haifa, Israel; 2 Department of Pediatric Endocrinology, Clalit Health Services, Haifa, Israel; 3 Research Authority, Clalit Health Care Organization, Carmel Medical Center, Haifa, Israel; 4 The Ruth & Bruce Rappaport Faculty of Medicine, Technion, Haifa, Israel; 5 Children's Endocrinology Consulting Center, Clalit Health Services, Afula, Israel; 6 Sleep Clinic, Clalit Health Services, Technion Faculty of Medicine, Haifa, Israel

**Keywords:** Sleep disorders, short stature, overweight, obesity, obstructive sleep apnea, growth hormone deficiency

## Abstract

**Background:**

Sleep disorders are common in childhood and are recognized as a significant public health concern, particularly due to their association with childhood obesity. However, their impact on growth disturbances has been only minimally explored. In the present study, we investigate the association between sleep disorders and linear growth and excess weight in children.

**Study Design:**

Data were collected retrospectively (January 2012–December 2022) from computerized medical records of 3,210 patients (newborn–18 years) diagnosed with sleep disorders who underwent sleep analysis at the Sleep Clinic of Carmel Medical Center. A control group (n =12,840), matched for age and ethnic background, was selected from the same healthcare database. Additionally, subgroup analyses were performed by sex and across three age groups.

**Results:**

A higher rate of short stature, defined as height at or below the 3rd percentile, was observed in the study group compared to controls (8.66% *vs*. 6.25%, p < 0.001). This difference was primarily seen in the 0–6 years age group (girls: OR 1.49, p = 0.003; boys: OR 1.57, p < 0.001). The diagnosis of short stature and growth hormone deficiency was documented in 10.1% and 2.1% of the study group, respectively, compared to 7.5% and 1.15% in the control group (p < 0.001 for both). Rates of overweight and obesity were higher among children in the study group compared to controls (overweight: 25.9% *vs*. 22.2%; obesity: 14.6% *vs*. 10.4%; p < 0.001). This difference was primarily observed in children aged 6–18 years, with the strongest effects seen in adolescent boys. Notably, children with obstructive sleep apnea had a significantly higher prevalence of short stature compared to those with other sleep disorders (13.4% *vs*. 7.7%; *p* < 0.001).

**Conclusions**

Our findings of a higher prevalence of growth disturbances and excess weight among children with sleep disorders highlight the importance of early recognition and timely intervention for pediatric sleep disorders. Such measures may improve sleep quality and daily functioning and potentially benefit growth and weight outcomes.

## Introduction

Sleep is a vital physiological requirement that is crucial for human life. It serves multiple restorative functions in the body and brain (1) and is regulated by biological mechanisms that involve the circadian rhythm (2). Specific neurotransmitter activities modulate the different stages of sleep; as a result, sleep deprivation can lead to alterations in hormone secretion. During sleep, growth hormone (GH), prolactin, and melatonin are released, and secretion of both cortisol and catecholamine decreases (3–5). Sleep deprivation or restriction results in increased evening cortisol and decreased anabolic hormones, including testosterone, GH, and insulin-like growth factor-1 (IGF-1) (1, 6).

Sleep is classified into non-rapid eye movement (NREM) and rapid eye movement (REM) stages. NREM sleep is composed of stages 1, 2, and 3; the formerly recognized stage 4 is currently combined with stage 3. Sleep is deepest in NREM stage 3, often referred to as slow-wave sleep (SWS) (7). GH is secreted as a series of pulses that occur mainly during sleep, with around three to six peaks per night. A GH surge is secreted during SWS. This sleep-onset GH pulse is caused by a surge of hypothalamic GH-releasing hormone which coincides with a circadian-dependent period of relative somatostatin disinhibition (6–9).

Sleep disorders are common in childhood and in the last few decades, they have been recognized as a major health problem, mainly related to the childhood obesity pandemic. However, only a limited number of studies have examined the relationship between sleep disorders and growth disturbances, with inconclusive findings. It is estimated that approximately 20%–50% of children have sleep disorders, including waking during the night, difficulty falling asleep, and difficulty sleeping alone. Sleep disorders are often underdiagnosed (7, 10).

Sleep disorders are more prevalent in children with neurodevelopmental disorders (11). Insufficient or poor quality of sleep has a pronounced impact on the child’s learning, behavior, mental state, and overall health (1, 2, 12–17). The most important potential mechanism associates sleep disorders, and short stature is reduced growth hormone secretion during deep sleep (1, 2, 4, 6, 8, 9). However, only a few studies—most with small sample sizes—have examined the association between sleep disorders and growth disturbances, with inconsistent findings. Verrillo et al. (6) studied a small group of children with GH deficiency (GHD) and age-matched controls using laboratory polysomnography. They found that children with GHD had significantly reduced total sleep time, sleep efficiency, movement time, and NREM sleep stage 2. Two studies one from Singapore involving 900 children (18), and another from North America with a sample of 23 children (19) reported a positive association between shorter sleep duration and short stature. In contrast, other studies failed to demonstrate any association between sleep duration and growth (20, 21).

The major reported medical comorbidity for sleep disorders in children and adolescents is obesity and consequently, metabolic syndrome (22). An association between shorter sleep duration and increased body mass index (BMI) has been reported in large population samples (23, 24). The potential mechanisms associating sleep disorders and weight change are complex and multifactorial. A bidirectional relationship between obesity and sleep disorders has been proposed: obesity predisposes individuals to sleep disorders—mainly obstructive sleep apnea (OSA), while poor quality and insufficient duration of sleep exacerbate weight gain through hormonal dysregulation, metabolic disturbances, and behavioral changes. The mechanism underlying the relationship between sleep disorders and obesity involves changes in appetite-regulating hormones reduced leptin and increased ghrelin secretion with a predominantly orexigenic effect. In addition, hyperactivation of the hypothalamic-pituitary-adrenal axis leads to increased cortisol secretion, resulting in increased appetite and expansion of visceral fat through the secretion of pro-inflammatory cytokines (25). Childhood obesity is linked to an increased need for healthcare services through various pathways, including sleep disorders (12, 16). Whether addressing sleep disorders can improve linear growth and reduce weight gain remains uncertain. In a previous study, we demonstrated variable changes in BMI z-score following adenotonsillectomy among adolescents with OSA (26).

In the present study, we examined the associations between sleep disorders, growth disturbances, and overweight/obesity by analyzing a cohort of 3,210 children diagnosed with sleep disorders in a specialized sleep clinic and comparing them to a large cohort of matched controls without sleep disorders.

## Methods

### Subjects

Data were collected retrospectively from computerized medical records of children and adolescents (ages 0–18 years) who underwent sleep analysis at the Sleep Clinic of Carmel Medical Center between January 2012 and December 2022 and were diagnosed with sleep disorders. We identified 3,210 patients with a diagnosis of sleep disorder who had auxologic measurements available within six months of diagnosis. Each case was matched to up to 20 controls of the same sex and age who had also auxologic measurements within six months of the matched case’s index date, yielding a total of 12,840 controls (median 5 controls per case). Control records were selected from the Clalit Health Services computerized database for the Haifa district, a comprehensive, high-quality, and carefully controlled database, with physicians inputting data during visits conducted in both hospital and community clinic settings. This database is highly reliable and includes records of all physician visits, prescribed medications, and over 98% of patient diagnoses (16, 26, 27). Collected data included demographic information, age, and auxologic measurements—weight, height, and BMI. Reported diagnoses in the individuals' medical files included short stature, GHD, obesity, overweight, hyperlipidemia, hypertension, fatty liver, and type 2 diabetes mellitus (T2DM).

### Auxologic data

Height and weight data were retrieved from the computerized medical records. The measurements used in this study were those closest to the index date of diagnosis, within a 6-month window post-diagnosis. For controls, the index date was defined as the diagnosis date of the matched case. Height and weight were converted to percentiles and standard deviations (SD) based on sex and age, according to the Centers for Disease Control and Prevention (CDC) year 2000 growth charts (28), which are routinely used in clinical practice in Israel. Short stature was defined as a height at or below the 3rd percentile at the time of the study. Overweight was defined as weight ≥85th percentile and <95th percentile, obesity was defined as weight ≥95th percentile. BMI was calculated by dividing weight in kilograms by the square of height in meters, with BMI percentiles derived from CDC charts (29). BMI values ≥85th percentile and <95th percentile were classified as overweight, and values ≥95th percentile were classified as obese.

### Sleep evaluation

Sleep analysis was conducted at a specialized pediatric sleep clinic at Carmel Medical Center. All patients aged 0–18 years who were diagnosed with sleep disorders between the years 2012 and 2022 were included. Diagnosed sleep disorders included sleep disordered breathing, insomnia (characterized by difficulties initiating and/or maintaining sleep, early morning awakening, and associated daytime functional complaints), parasomnias (such as sleep terrors, sleepwalking, and nightmares), restless legs syndrome, narcolepsy, and hypersomnia. For the diagnosis of sleep disordered breathing, children underwent overnight polysomnography. OSA was diagnosed when the apnea-hypopnea index exceeded two events per hour of sleep. For other sleep disorders, diagnoses were primarily based on clinical history and characteristics obtained during sleep clinic visits.

### Statistical analysis

All analyses were performed using R software, version 4.3.1 (Vienna, Austria). In the univariate analysis, demographic and clinical parameters were compared between cases and controls. Continuous variables were summarized as means ± SD and compared using the two-sample *t*-test. Categorical variables were summarized as percentages and compared using Pearson’s chi-square test or Fisher’s exact test, as appropriate. The distributional properties of weight, BMI, and height percentiles were evaluated. To assess the association between sleep disorders and anthropometric outcomes, we analyzed six binary endpoints: height <3rd percentile, height <5th percentile, weight >85th percentile, weight >95th percentile, BMI >85th percentile, and BMI >95th percentile. Because the data were frequency-matched, unconditional logistic regression models were used for each outcome, including case–control status, sex, and age group (0–6, 6–12, 12–18 years), as well as all two- and three-way interaction terms. This approach allowed us to evaluate whether the odds of being equal, below or above each anthropometric threshold differed between groups. The matched nature of the controls was accounted for by calculating cluster-robust standard errors using the original matched set identifier. For interpretability, odds ratios (ORs) with 95% confidence intervals (CIs) were reported for the case–control comparison within each sex–age stratum. Statistical significance was defined as p < 0.05.

The study was conducted in accordance with the principles of the Declaration of Helsinki, and ethical approval was obtained from the Institutional Review Board of Carmel Medical Center.

## Results

The baseline characteristics of the 3,210 children diagnosed with sleep disorders (study group) and 12,840 children in the control group are summarized in **Table 1**. Their height parameter data are presented in **Table 2**. The mean height for age was in the 45.1^th^ percentile in the study group compared to the 46.9^th^ percentile in the control group (*p* = 0.001). In the study group, 8.66% had a height at or below the 3^rd^ percentile, compared to 6.25% in the control group (p<0.001). When we subdivided the cohort according to age and sex, the difference between groups was significant only in the 0–6 years age group (girls: OR 1.49, p = 0.003; boys: OR 1.57, p < 0.001). Similar findings were observed for height at or below the 5th percentile (girls: OR 1.37, p = 0.007; boys: OR 1.48, p < 0.001) (**Table 3**). Among the 3,210 patients with sleep disorders, 16.3% had OSA. Both the OSA group and the group with other sleep disorders had higher rates of males. The mean age at diagnosis of children with OSA was significantly younger than that of those diagnosed with other types of sleep disorders. Children with OSA had a higher rate of growth retardation compared to controls (13.4 *vs*. 6.3, *p* < 0.001) and compared to non-OSA patients (**Table 4**). A higher prevalence of GHD was reported in the OSA group compared to the non-OSA group (**Table 4**).

**Table 1:** Demographic characteristics of the study group and controls.

Characteristic	Study group	Control group	*p* value
Total no. (%)	3,210	12,840	
Male	1,926 (60%)	7,817 (61%)	0.4
Female	1,284 (40%)	5,023 (39%)	
Age subgroups (y)			<0.001
0-6	2280 (71%)	9991 (77.8)	
6-12	616 (19.2%)	1692 (13.2%)	
12-18	314 (9.8%)	1157 (9%)	
Age at diagnosis (y) Mean ± SD (Range)	5.02 ± 4.0 (0–18)	4.75 ± 4.0 (0–18)	0.071
Origin			
Jewish	2,162 (72%)	7,968 (72.32%)	
Arab-Muslim	609 (20.3%)	2,354 (21.4%)	
Arab-Christian	49 (1.6%)	187 (1.7%)	
Druze	181 (6.0%)	509 (4.6%)	

Y, years.

**Table 2:** Height parameters of the study group and controls.

Characteristic	Study group	Control group	*p* value
No.	3210	12.840	
Height percentile Mean ± SD (Range)	45.1 ± 30.5 (3.0–97)	46.9 ± 29.4 (3.0–97)	0.001
Height <3^rd^ percentile No (%)	278 (8.66%)	803 (6.25%)	<0.001
Height <5^th^ percentile No. (%)	378 (11.8%)	1,166 (9%)	<0.001
Diagnosis of short stature No. (%)	322 (10.1%)	964 (7.5%)	<0.001
Diagnosis of GHD No. (%)	67 (2.1%)	148 (1.15%)	<0.001

**Table 3:** Odds ratio for short stature in the study group compared with controls by sex and age group.

Sex	Age (y)	Outcome	Study group (No.)	Controls (No.)	Study group No. (%)	Control group No. (%)	OR (95% CI)	p-value
Female	0-6	Height<3rd percentile	867	3789	82 (9.5%)	249 (6.6%)	1.49 (1.15–1.92)	0.003
	6-12		271	742	17 (6.3%)	32 (4.3%)	1.48 (0.83–2.65)	0.18
	12-18		146	495	11 (7.5%)	32 (6.5%)	1.18 (0.58–2.41)	0.651
Male	0-6		1413	6202	141 (10%)	408 (6.6%)	1.57 (1.29–1.92)	<0.001
	6-12		345	950	17 (4.9%)	42 (4.4%)	1.12 (0.62–2.04)	0.71
	12-18		168	662	10 (6%)	40 (6%)	0.98 (0.5-1.95)	0.963
Female	0-6	Height<5th percentile	867	3789	109 (12.6%)	359 (9.5%)	1.37 (1.09–1.73)	0.007
	6-12		271	742	25 (9.2%)	49 (6.6%)	1.44 (0.86–2.40)	0.165
	12-18		146	495	14 (9.6%)	47 (9.5%)	1.10 (0.54–1.9)	0.973
Male	0-6		1413	6202	191 (13.5%)	593 (9.6%)	1.48 (1.24–1.76)	<0.001
	6-12		345	950	27 (7.8%)	56(5.9%)	1.36 (0.84–2.19)	0.214
	12-18		168	662	12 (7.1%)	62 (9.4%)	0.74 (0.39–1.41)	0.367

Study group (%): Number of cases and percentage of the subgroup with the outcome (calculated as *N case with outcome ÷ total N case × 100*). Controls (%): Number of controls and percentage of the subgroup with the outcome (calculated as *N control with outcome ÷ total N control × 100*).

**Table 4:** Comparison of growth parameters between OSA and non-OSA patients in the study group.

Characteristic	OSA	Non-OSA	*p* value
No.	524	2,686	
Male No. (%)	336 (64.1%)	1,590 (59.2%)	0.035
Age at diagnosis (y) Mean ± SD	3.91 ± 2.7	5.23 ± 4.2	<0.001
Height <3^rd^ percentile No (%)	70 (13.4%)	208 (7.7%)	<0.001
Height<5^th^ percentile No. (%)	84 (16%)	294 (11%)	<0.001
Diagnosis of GHD No. (%)	18 (3.4%)	49 (1.8%)	0.019

GHD, growth hormone deficiency, SA, obstructive sleep apnea.

Weight and BMI parameters are shown in **Table 5**. Higher rates of overweight and obesity were observed in the study group compared to controls using weight percentiles (≥85^th^: 18.5% *vs*. 14.6%, p < 0.001; ≥95^th^; 8.97% *vs*. 4.7%, p < 0.001) and BMI percentiles (overweight: 25.9% *vs*. 22.2%, p < 0.001; obesity: 14.6% *vs*. 10.4%, p < 0.001). These differences were significant mainly between the ages of 6–18 years among both girls and boys (**Table 6**). Weight ≥85^th^ percentile was higher in the study group compared to controls in girls aged 6–12 and 12–18 years (OR 1.62, p = 0.002; OR 1.62, p = 0.021, respectively) and in boys (OR 1.90, p < 0.001; OR 2.76, p < 0.001, respectively). Similarly, weight >95^th^ percentile was elevated in girls aged 6–12 and 12–18 years (OR 1.61, p = 0.012; OR 2.69, p < 0.001, respectively) and in boys (OR 2.44, p < 0.001; OR 4.90, p < 0.001, respectively). The same results were found in BMI parameters. For girls BMI ≥85^th^ percentile at 6–12 and 12–18 years (OR 1.64, p< 0.001; OR 1.86, *p* = 0.001, respectively), and boys (OR 1.68, *p* < 0.001; OR 2.39, p< 0.001, respectively. BMI >95^th^ percentile for girls (OR 1.7, *p* < 0.001, OR 2.30, p< 0.001, respectively) and for boys (OR 2.04, p<0.001, OR, 3.3, p<0.001, respectively) (**Table 6**).

**Table 5:** Weight and BMI parameters in the study group compared to controls.

Parameter	Study group	Controls	*p* value
No.	3,210	12,840	
BMI (percentile)	55.2 ± 32.5	54.5 ± 30.1	0.065
Weight ≥85^th^ No. (%)	593 (18.5%)	1,871 (14.6%)	<0.001
Weight ≥95^th^ No. (%)	288 (8.97%)	604 (4.7%)	<0.001
BMI ≥85^th^ No. ('%)	831 (25.9%)	2,855 (22.2%)	<0.001
BMI ≥95^th^ percentile, No. (%)	468 (14.6%)	1,331 (10.4%)	<0.001
Diagnosis of obesity, No. (%)	883 (27.6%)	2,745 (21.4%)	<0.001

**Table 6:** Odds ratios for BMI percentiles in children with sleep disorders compared with controls, by sex and age group.

Sex	Age (y)	Outcome	Study group (No.)	Controls (No.)	Study group No. (%)	Control group No. (%)	OR (95% CI)	p-value
Female	0-6	Weight>85^th^ percentile	867	3789	108 (12.5%)	466 (12.3%)	1.01 (0.81–1.27)	0.897
	6-12		271	742	79 (29.2%)	150 (20.2%)	1.62 (1.20–2.20	0.002
	12-18		146	495	48 (32.9%)	115 (23.2%)	1.62 (1.08–2.44)	0.021
Male	0-6		1413	6202	191 (13.5%)	844 (13.6%)	0.99 (0.84–1.18)	0.929
	6-12		345	950	99 (28.7%)	166 (17.5%)	1.90 (1.45–2.50)	<0.001
	12-18		168	662	68 (40.5%)	131 (19.8%)	2.76 (1.92-3.95)	<0.001
Female	0-6	Weight>95^th^ percentile	867	3789	37 (4.3%)	128 (3.4%)	1.28 (0.89–1.84)	0.192
	6-12		271	742	40 (14.8%)	72 (9.7%)	1.61 (1.11–2.34)	0.012
	12-18		146	495	35 (24%)	52 (10.5%)	2.69 (1.66–4.36)	<0.001
Male	0-6		1413	6202	75 (5.3%)	236 (3.8%)	1.42 (1.08–1.85)	0.011
	6-12		345	950	56 (16.2%)	70 (7.4%)	2.44 (1.70–3.49)	<0.001
	12-18		168	662	45(26.8%)	46 (6.9%)	4.90 (3.23–7.44)	<0.001
Female	0-6	BMI>85^th^ percentile	867	3789	162 (18.7%)	741 (19.6%)	0.95 (0.78–1.14)	0.554
	6-12		271	742	101 (37.3%)	197 (26.5%)	1.64 (1.25–2.16)	<0.001
	12-18		146	495	66 (45.2%)	152 (30.7%)	1.86 (1.27–2.73)	0.001
Male	0-6		1413	6202	300 (21.2%)	1347 (21.7%)	0.97 (0.84–1.12)	0.69
	6-12		345	950	118 (34.2%)	224 (23.6%)	1.68 (1.31–2.16)	<0.001
	12-18		168	662	84 (50%)	195 (29.5%)	2.39 (1.66–3.45)	<0.001
Female	0-6	BMI>95^th^ percentile	867	3789	76 (8.8%)	306 (8.1%)	1.09 (0.84–1.42)	0.5
	6-12		271	742	65 (24%)	116 (15.6%)	1.70 (1.24–2.33	<0.001
	12-18		146	495	49 (33.6%)	89 (18%)	2.30 (1.56–3.40)	<0.001
Male	0-6		1413	6202	139 (9.8%)	604 (9.7%)	1.01 (0.83–1.23)	0.91
	6-12		345	950	75 (21.7%)	114 (12%)	2.04 (1.51–2.75)	<0.001
	12-18		168	662	64 (38.1%)	104 (15.7%)	3.3 (2.29–4.76)	<0.001

Study group (%): Number of cases and percentage of the subgroup with the outcome (calculated as *N case with outcome ÷ total N case × 100*). Controls (%): Number of controls and percentage of the subgroup with the outcome (calculated as *N control with outcome ÷ total N control × 100*).

Increased prevalence of obesity related complications, including cardiometabolic complications such as hypertension, hypertriglyceridemia, hyperlipidemia, fatty liver, and T2DM, was found in the study group (**Table 7**). These complications were observed predominantly in the 12–18 age group with statistically significant differences: hyperlipidemia (OR 1.60, p=0.031), Hypertriglyceridemia (OR 3.46, p=0.003), Fatty liver (OR 3.58, p < 0.001), and T2DM (OR 2.50, p = 0.027).

**Table 7:** Other diagnoses in the study group and controls.

Parameter	Study group	Controls	*p* value
No. (%)	3,210	12,840	
Hyperlipidemia	100 (3.1%)	200 (1.6%)	<0.001
Hypertriglyceridemia	18 (0.6%)	39 (0.3%)	0.028
Hypertension	58 (1.8%)	114 (0.9%)	<0.001
Fatty liver	49 (1.5%)	64 (0.5%)	<0.001
T2DM	20 (0.63%)	47 (0.37%)	0.042
Suicide attempt	17 (0.5%)	33 (0.3%)	0.013
Convulsive disorder	132 (4.1%)	188 (1.5%)	<0.001

Diagnoses were retrieved from the individuals’ computerized files. T2DM, type 2 diabetes.

## Discussion

Significant growth impairment was observed in children with sleep disorders compared to the control group. Short stature was diagnosed in a significantly greater number of children in the study group, and a higher rate of GHD was reported among children with sleep disturbances. In a subgroup analysis stratified by age and sex, we observed differential effects of sleep disorders on linear growth. Short stature was most pronounced in early childhood, with both boys and girls aged 0–6 years showing significantly increased odds of being at or below the 3^rd^ and 5^th^ height percentiles.

GH secretion mainly occurs during deep sleep (SWS), yet limited studies have examined the link between sleep disorders and growth disturbances (2). Research shows that children with GHD have reduced total sleep duration, sleep efficiency, and time in stage 2 NREM sleep (2, 6), while GH therapy has been shown to enhance sleep efficiency (30). Guilhaume et al. (31) found through polysomnography that children with psychosocial short stature had significant deficits in stage 4 NREM sleep and shorter SWS compared to controls, with improvements noted in sleep quality and increased duration of stage 4 sleep during a growth-recovery phase (31).

Disruptions in sleep architecture—such as reduced SWS or frequent awakening—may interfere with normal endocrine function, including GH secretion, thereby contributing to suboptimal growth outcomes (1, 2, 4, 6, 8, 9). Our findings are novel, particularly in demonstrating that sleep disorders are associated with impaired linear growth mainly in early childhood. This may be explained partially by the fact that linear growth during this period is highly dependent on GH which is secreted primarily during deep sleep. Disruptions in sleep architecture at this critical developmental stage may therefore impair GH release and negatively affect growth. In older children, growth is influenced by additional factors, such as puberty-related hormonal changes. Another possible explanation for the growth impairment observed among children aged 0–6 years with sleep disorders relates to the high prevalence of OSA secondary to adenoid and tonsillar hypertrophy in this age group. Since our study is cross-sectional, we were not able to determine whether these patients experienced catch-up growth later in life. While previous studies have shown that both the quantity and quality of sleep are essential for healthy somatic development (6, 18, 19, 32), our study demonstrates associations between sleep disorders and growth; given its observational design, causality cannot be inferred. It has been proposed that ghrelin, which binds to the hypothalamic GH secretagogue receptor that plays an important role in regulating GH secretion and appetite control, may underline the effect of abnormal sleep on growth and weight gain, given ghrelin’s interaction with circadian rhythms (32).

The prevalence of sleep disorders was higher among boys, with the proportion of males being even greater in the OSA group. This finding is supported by evidence indicating that certain sleep disturbances—particularly OSA—are more common in boys than girls (33), especially after puberty (34). In younger children, OSA is most often caused by hypertrophic tonsils and adenoids, and its prevalence is similar between the sexes. However, during puberty, the male airway becomes more susceptible to collapse, contributing to the increased prevalence of OSA in adolescent boys (34). In addition, neurodevelopmental conditions such as attention deficit hyperactive disorder (which are more common in boys) are often associated with sleep disturbances (14, 35).

Patients with OSA were significantly younger at diagnosis than those with other types of sleep disorders (**Table 4**). This difference may be explained by the fact that OSA often presents with noticeably disruptive symptoms, prompting earlier medical evaluation. In contrast, other sleep disorders may have a more subtle or gradual onset, which can result in delayed recognition and diagnosis. It has been shown that OSA is more common in 2- to 6-year-olds (36).

Children with OSA had a higher rate of growth retardation and GHD compared to controls and compared to non-OSA patients. Our findings align with the albeit limited studies indicating an association between sleep disordered breathing in children and delayed growth (37, 38), the underlying mechanisms of which remain to be elucidated (1). Obese patients with OSA show a reduction in both spontaneous and stimulated GH secretion coupled with reduced IGF-1 and impaired peripheral sensitivity to GH. These endocrine abnormalities are more pronounced than in non-apneic obese subjects, and are likely due to the effects of hypoxia and sleep fragmentation on hormones secretory pattern. The disruption in GH/IGF-1 axis activity may be responsible, at least in part, for metabolic alterations, which are common in children with OSA and increase the risk of cardiovascular events, as well as mortality (39). Interestingly, we found that 6.25% of children in the control group had a short stature, defined as height at or below the 3^rd^ percentile. We assume that the relatively high rate of short stature among the controls may be attributed to the use of CDC growth charts, which reflect the U.S. population and may not be entirely suitable for the Israeli population. Nevertheless, the prevalence of short stature was significantly higher among children with sleep disorders, reinforcing the observed association between sleep disturbances and impaired growth.

Another major finding, we obtained in our study is that patients with sleep disorders were found to have higher prevalence of overweight and obesity and a higher prevalence of cardiometabolic complications—hyperlipidemia, hypertension, fatty liver, and T2DM, aligning with other studies indicating these tendencies (22–25). Interestingly, these findings were predominantly observed in children aged 6–18 years in both sexes, with the highest rates seen in adolescent boys. We previously showed that children with OSA have severity-dependent overnight deterioration of endothelial function, an early risk marker for cardiovascular disease using the peripheral arterial tonometry technique. This may partially explain the tendency for cardiovascular consequences in patients with OSA. The intermittent increases in upper airway resistance during sleep in OSA result in oxyhemoglobin desaturation, increased carbon dioxide levels, and activation of the sympathetic nervous system, resulting in endothelial dysfunction (17).

It is suggested that sleep deprivation acts directly on the risk of being overweight/obesity through its role in sympathetic activation, increased cortisol secretion, and increased levels of interleukin and tumor necrosis factor via activation of the inflammatory cascade. These result in insulin resistance and pancreatic β-cell dysfunction, which can lead to glucose intolerance, weight gain, and the development of T2DM (25, 40). The alteration in appetite-regulating hormones, i.e., reduced leptin and increased ghrelin secretion, leads to a predominantly orexigenic effect (25, 41). Our results indicate an increased risk of excess weight in late childhood and adolescence among patients with sleep disorders. This may be partly explained by the fact that, from mid-childhood onward, children tend to obtain fewer hours of sleep. Indeed, longitudinal analyses indicate a global decline in children’s sleep duration over the past century (42), and reduced sleep may play an important role in the current obesity pandemic (24, 41, 43). One explanation for this sex-specific vulnerability could be the higher proportion of short sleepers on weekdays and weekends among boys compared to girls (44). A limitation of our study is its retrospective data-based design, which does not allow for the exploration of underlying mechanisms or causal relationships. In addition, detailed information on sleep-stage distribution was not available for participants who had undergone polysomnography. Another limitation of our study is that, given its design, we could not exclude all potential underlying conditions for each participant. Notably, the study group had a higher prevalence of convulsive disorders, which may have influenced growth parameters. Additionally, potential biases in auxologic measurements may be occurred due to the use of different devices and examiners. As our data are cross-sectional and reflect only the time point of sleep disorder diagnosis, we were unable to assess the impact of treatment on growth and weight outcomes. Nevertheless, the strong associations identified between sleep disorders and growth parameters highlight the need for further research to explore causality and underlying physiological mechanisms.

## Conclusions

Our findings of a higher prevalence of growth disturbances and excess weight among children with sleep disorders highlight the importance of early recognition and timely intervention for pediatric sleep disorders. Such measures may improve sleep quality and daily functioning and potentially benefit growth and weight outcomes.

## Data Availability

The original contributions presented in the study are included in the article/supplementary material. Further inquiries can be directed to the corresponding author.
